# Prevalence of Sleep Disturbance and Potential Associated Factors among Medical Students from Mashhad, Iran

**DOI:** 10.1155/2020/4603830

**Published:** 2020-07-05

**Authors:** Ahmad Janatmakan Amiri, Negar Morovatdar, Atefeh Soltanifar, Ramin Rezaee

**Affiliations:** ^1^Clinical Research Development Unit, Imam Reza Hospital, Faculty of Medicine, Mashhad University of Medical Sciences, Mashhad, Iran; ^2^Psychiatry and Behavioral Sciences Research Center, Mashhad University of Medical Sciences, Mashhad, Iran; ^3^Department of Chemical Engineering, Environmental Engineering Laboratory, Aristotle University of Thessaloniki, Thessaloniki, Greece

## Abstract

**Methods:**

In this cross-sectional study, 315 medical students chosen by stratified random sampling participated in the academic year 2018-2019. The Pittsburgh Instrument and DASS-21 questionnaire were used to evaluate sleep quality and anxiety, depression, and stress, respectively. Also, demographic, educational, and socioeconomic information was collected. SPSS 16 software was used for data analysis.

**Results:**

Out of 300 students who completed the questionnaires, 165 (55%) were male, with a mean age of 21.94 ± 2.28 years old. The prevalence of poor sleep quality was 51.3%. We did not find significant associations among age, sex, and poor sleep quality. Concurrent psychological symptoms such as stress, depression, and anxiety were significantly associated with sleep disorders. After adjusting variables in the multivariable regression model, depression (OR = 2.81, 95% CI: 1.35-5.87; *p* = 0.006) and the number of hours spent on using smartphones in 24 hours (OR = 1.13, 95% CI: 1.02-1.25; *p* = 0.01) were significantly associated with poor sleep quality among medical students.

**Conclusion:**

The prevalence of poor sleep quality among medical students was high, and we found that increased use of smartphones during the day and depression were associated with sleep disorders.

## 1. Introduction

Sleep is an essential physiological process of life. Sleep disturbance negatively affects our physical [1] and psychological health [2]. Approximately one-third of adults report some forms of sleep problems. In the recent years, sleepiness and sleep problems are endemic in the population [3]. Medical students are a subgroup of the general population who appear to be especially vulnerable to poor sleep quality, perhaps due to the long duration and high intensity of study, clinical duties that include overnight on-call duties, work that can be emotionally challenging, and lifestyle choices [4]. Some studies estimated that the prevalence of sleep disorders ranges between 15 and 42% in general population [5, 6], and a report showed that the prevalence of such conditions in Iranian medical students was 30% [7]. Several studies demonstrated that age, sex, socioeconomic status, life habits, smartphone overuse, and psychological are risk factors for sleep disturbance [6, 8, 9]. Sleep disturbance can affect functions such as cognitive and psychomotor performance which are vital for medical students and may endanger patients' life and influence the overall performance of the health care system [10]. It is important to know the prevalence of such conditions and potential related factors for detection and treatment of such problems in medical students. Mashhad, the second largest city of Iran, is located in the northeast of Iran. The aim of the current study was to determine the prevalence of sleep disturbance in medical students of Mashhad University of Medical Sciences, Mashhad, Iran, and potential associated factors.

## 2. Methods

### 2.1. Study Population and Sampling

The study sample consisted of 315 medical students (19 to 25 years old) of the first to seventh year during the academic year 2018-2019. We used a stratified random sampling method. Medical students were recruited during their rest times between classes. First, the objective of the study was described for the students. After obtaining verbal consent, those who agreed to participate in the study received a questionnaire. Sample size was calculated based on the Cochran formula for limited population with *α* = 0.05 and power = 0.8. This study was funded and approved by Mashhad University of Medical Sciences (No. 961022).

### 2.2. Instrument

We used a self-administered questionnaire developed based on Depression, Anxiety, and Stress Scales (DASS-21) and Pittsburgh Sleep Quality Index (PSQI). The DASS-21 questionnaire consists of 21 questions in three different domains of depression. This questionnaire assesses anxiety and stress, by seven questions for each. The Likert scale ranging from 0 (disagree) to 3 (agree) was applied for each question. Based on total score and classification of the questionnaire, the disorder was defined as normal, mild, moderate, severe, and extremely severe [11]. The validity and reliability of the Persian version of this questionnaire were previously reported [12]. The Persian version of the PSQI questionnaire which includes 34 closed questions was valid and reliable. The PSQI is a self-rating questionnaire with 19 questions and seven component scores: sleep quality, sleep latency, sleep duration, habitual sleep efficiency, sleep disturbances, use of sleeping medication, and daytime dysfunction. It was originally designed as a simple and valid instrument for use under diverse clinical conditions [13]. The 19 self-rated questions are grouped to form seven component scores. Each component score is rated from 0 to 3 (0, not in the past month; 1, less than once per week; 2, once or twice per week; and 3, three or more times per week). The global score ranges from 0 to 21, with higher scores indicating poorer sleep quality. The demographic characteristics included age, gender, and marital status.

### 2.3. Statistical Analysis

Frequency tables are used to present the distribution of nominal variables. Numeric variables are given as mean and standard deviation (SD). We used *χ*^2^, Student's *t*-test, one-way ANOVA, and Tukey's test as the *pot hoc* test, for analyzing data. Logistic regression was used to estimate odds ratio (OR) with its 95% CI for associated factors. Data was analyzed by SPSS version 16.0 (SPSS, Inc., Chicago, IL). The significance level was set at *p* < 0.05.

## 3. Results

From 310 students recruited in this study, 300 students completed questionnaires and they were included in the analysis. The mean age of the study population was 21.94 ± 2.28 years old, and 165 (55%) were male. Among them, 46 (15.3%) were married and 117 (39%) were living in dorms. In our study population, 5.7% had a history of sleep disorders and needed to use medicines. In this regard, 16% of students were using hypnotic drugs to have night sleeping. Also, 280 (93.3%) of them were using smartphones before going to sleep in the bed. The mean duration of smartphone use was 4.48 (2.53) hours during 24 hours.

Based on the PSQI questionnaire, 154 (51.3%) students who participated in our study had sleep problems. Frequency of sleep disturbance was not significantly different between the sex groups (53% in males vs. 48.9% in females; *p* = 0.44). We did not find any association between marital status and sleep disturbance (Table 1).

We found that the average number of hours spent on using smartphones was significantly higher in students with sleep disorders compared to ones without this problem (4.89 ± 2.84 vs. 4.05 ± 2.09; *p* = 0.001).

According to the DASS-21 questionnaire, 35% of the students had anxiety, 24% had stress, and 35% had depression. Also, we found that depression, anxiety, and stress were significantly associated with sleep disorders (Table 2).

Among different factors that were evaluated for potential association with sleep disorders, after adjusting in the multivariable regression model, depression (OR = 2.81, 95% CI: 1.35-5.87; *p* = 0.006) and the number of hours spent on using smartphones in 24 hours (OR = 1.13, 95% CI: 1.02-1.25; *p* = 0.01) were significantly associated with sleep disorders.

## 4. Discussion

Our study showed that half of the medical students who participated in the present study had poor sleep quality. In other studies, the prevalence of poor sleep quality varied from 16% in Malaysian medical students [14] to 40.6% in Iranian [15] and Lithuanian medical students [16] and 57% in Nigerian medical students [17]. Different studies compared the prevalence of this problem between medical and nonmedical students and found that medical students had the highest prevalence of such condition compared to other students [10, 16]. This difference might be due to heavier academic load and their different lifestyles [10]. Medical students are a subgroup of the general population who are susceptible to poor sleep, for heavy clinical duties, long duration of study, on-call duties, challenging workplace, and different lifestyle [4].

We did not find any association between sex and sleep quality in our study population. Our results were inconsistent with other studies conducted in Hong Kong and Pakistan that found higher rate of poor sleep quality in female students compared to males [18, 19]. The association between female gender and poor sleep quality might be partly explained by higher frequency of sleep disorders and disturbances among females in general population [20]. Also, sociocultural norms of societies, like higher rates of involvement in household duties in females, may also account for such gender differences.

Higher age was reported as a risk factor for sleep disturbance and sleep dissatisfaction in general population [21]. However, in medical students, sleep problems are common even in young medical students [22]. We did not find any association between age and poor sleep quality, probably due to small variation in age as the participants were all from the same age group.

We found that 35% of our students suffer from anxiety or depression and 24% from stress. There is a correlation between psychiatric illnesses especially anxiety and mood disorders and sleep disorders [23]. It should be noted that psychiatric disorders might be caused by insomnia [24]. Also, a Brazilian study revealed an association between psychiatric disorders and insomnia and authors believed that sleep disorder assessment might be a good tool for identification of other psychological issues that may underlie sleep disorders [25]. In Chinese medical students, there was a significant association between sleep quality and depression or anxiety [26]. Another factor that was found to be associated with poor sleep quality in our study was overuse of smartphones by medical students. In this regard, our results were similar to those reported from Iran [27] and India [28] for medical students.

Our study had some limitations. The design of our study was cross-sectional and the phenomenon of inverse causality needs to be considered for an association between factors such as depression and overuse of smartphones with poor sleep quality. Nevertheless, as there are few studies that evaluated sleep disturbance among medical students, the results of this study could be considered a basic step for development of preventive measures for early detection and treatment of such problems in students.

## 5. Conclusion

The present study found a high prevalence of poor sleep quality among medical students. Factors associated with increased risk of poor sleep quality were depression and overuse of smartphones.

## Figures and Tables

**Table 1:** Compare demographic characteristics between the two groups.

	With sleep disturbance (*N* = 154)	Without sleep disturbance (*N* = 146)	*p*
Sex (male)	88 (57%)	77 (52.7%)	0.44
Age	22.05 (2.49)	21.82 (2.49)	0.20
Married	21 (13.6%)	25 (17.12%)	0.40
Living place			
Dormitory	60 (38.9%)	57 (39%)	0.99
Owned house	39 (25.3%)	36 (24.6%)	
With family member	55 (35.7%)	53 (36.3%)	
Family member (number)	4.62 (1.32)	4.41 (1.34)	0.44

**Table 2:** Comparing psychological and behavioral characteristics between the two groups.

	With sleep disturbance (*N* = 154)	Without sleep disturbance (*N* = 146)	*p*
Depression problems	71 (46.1%)	33 (22.6%)	0.001
Anxiety problems	64 (41.5%)	42 (28.7%)	0.02
Stress problems	57 (37%)	16 (10.9%)	0.001
Time spent on mobile use in 24 hours	4.89 (2.84)	4.05 (2.09)	0.001
Mobile use before getting to the bed	143 (92.8%)	137 (93.8%)	0.73
History of failing the exam	46 (29.8%),	44 (30%)	0.96

## Data Availability

Mashhad University of Medical Sciences holds the copyright of the SPSS file that contains data obtained in this study, and the file cannot be made freely available.
